# A microfluidic biosensor for the diagnosis of chronic wasting disease

**DOI:** 10.1038/s41378-023-00569-1

**Published:** 2023-08-21

**Authors:** Sura A. Muhsin, Amjed Abdullah, Estela kobashigawa, Muthana Al-Amidie, Sherri Russell, Michael Z. Zhang, Shuping Zhang, Mahmoud Almasri

**Affiliations:** 1 University of Missouri–Columbia, Electrical Engineering and Computer Science, Columbia, MO USA; 2 University of Missouri–Columbia, College of Veterinary Medicine, Veterinary Medical Diagnostic Laboratory, Columbia, MO USA; 3 Missouri Department of Conservation, Columbia, MO USA

**Keywords:** Electrical and electronic engineering, Nanoparticles

## Abstract

Cervids are affected by a neurologic disease that is always fatal to individuals and has population effects. This disease is called chronic wasting disease (CWD) and is caused by a misfolded prion protein. The disease is transmitted via contact with contaminated body fluids and tissue or exposure to the environment, such as drinking water or food. Current CWD diagnosis depends on ELISA screening of cervid lymph nodes and subsequent immunohistochemistry (IHC) confirmation of ELISA-positive results. The disease has proven to be difficult to control in part because of sensitivity and specificity issues with the current test regimen. We have investigated an accurate, rapid, and low-cost microfluidic microelectromechanical system (MEMS) biosensing device for the detection of CWD pathologic prions in retropharyngeal lymph nodes (RLNs), which is the current standard type of CWD diagnostic sample. The device consists of three novel regions for concentrating, trapping, and detecting the prion. The detection region includes an array of electrodes coated with a monoclonal antibody against pathologic prions. The experimental conditions were optimized using an engineered prion control antigen. Testing could be completed in less than 1 hour with high sensitivity and selectivity. The biosensor detected the engineered prion antigen at a 1:24 dilution, while ELISA detected the same antigen at a 1:8 dilution. The relative limit of detection (rLOD) of the biosensor was a 1:1000 dilution of a known strong positive RLN sample, whereas ELISA showed a rLOD of 1:100 dilution. Thus, the biosensor was 10 times more sensitive than ELISA, which is the currently approved CWD diagnostic test. The biosensor’s specificity and selectivity were confirmed using known negative RPLN samples, a negative control antibody (monoclonal antibody against bovine coronavirus BCV), and two negative control antigens (bluetongue virus and Epizootic hemorrhagic disease virus). The biosensor’s ability to detect pathogenic prions was verified by testing proteinase-digested positive RLN samples.

## Introduction

Chronic wasting disease (CWD) is a fatal, highly contagious, neurodegenerative disease affecting cervid species such as white-tailed deer (*Odocoileus virginianus*), mule deer (*Odocoileus hemionus*), and Rocky Mountain elk (*Cervus elaphus nelsoni*) in North America^1,2^. CWD is a disease called transmissible spongiform encephalopathy (TSE). CWD is unique in that it is the only TSE known to free-ranging populations, causing it to be particularly challenging to manage its spread and prevalence^3,4^. CWD currently affects 26 states in the United States as well as 3 Canadian provinces. The specific number may vary depending on the source and the time of the information, as the distribution of the disease can change over time^5^. Due to the ever-expanding spread of CWD, increased sensitivity of the detection methods is required to effectively manage and minimize the amount of prion in the environment^6^.

TSEs are classified as prion diseases due to their characteristic misfolding of the normal cellular glycoprotein (PrP^C^)^7^. Under current models, TSEs act on the naïve PrP^C^ protein by changing the composition of α-helices to β-sheets^8^, providing the PrP^SC^ molecule resistance to protease digestion. This causes the protein to refold to the infectious PrP^SC^, which when taken up by a host, is transported and propagated within the host, eventually infects the nervous system and leads to an increasing conversion of PrP^C^ to PrP^SC,^^8,9^. When enough of the protein is converted, clinical signs begin to show, consisting of physiologic and behavioral abnormalities, such as altered stance with a lowered head, excess salivation due to difficulty swallowing, and a general lack of awareness^6^. Concurrently, as PrP^SC^ molecules propagate and continue to recruit normal cellular proteins in the host system, the disease progresses^8^. Once a host sheds the prion agent, horizontal transmissibility persists in the environment through various vectors and/or reservoirs, although the stability of these reservoirs remains unclear^10^.

The gaps in information regarding prion stability and persistence prevent the proper implementation of specifically curated disease management strategies. Recent studies suggest that prions appear to bind sands, soils, and clays, which ruminants, such as white-tailed deer, commonly ingest while feeding^11^. Once bound, the bonds between the prion and the environmental agent via the net positively charged N-terminus of the prion and the negatively charged surface of the mineral do not readily dissociate and remain highly infectious^11^. To support these findings, previous studies have speculated that once prions have been shed in an environment, they will likely persist for years^2^. According to Saunders^2^, decontamination techniques exist but may not always be practical and are largely limited by detection methods. As such, limiting the amount of prion in the environment is the most viable management technique until an effective decontamination method can be developed.

Considering CWD’s long incubation period, which can last years before becoming clinical and causes it to be particularly insidious and difficult to manage^12^. Termination earlier in the replication process will lead to fewer infectious prions being shed in the environment. Various approaches to controlling CWD have emphasized the minimization of the risks associated with insufficient knowledge of the possible outcomes^13,14^. Approaches, such as proactive hunting surveillance and culling, conducted by government agencies have been shown to be effective in managing the prevalence of CWD^14,15^. These management techniques act on CWD by combating the build-up of infectious material in the environmental reservoirs^15^. This provides a protective buffer to populations and areas not yet impacted by CWD by removing individuals from known endemic areas and preventing them from interacting with naïve individuals. These strategies, hunting surveillance and targeted culling, are naturally reactionary. While these strategies have proven effective at maintaining prevalence, it is clear that preemptive strategies would be appreciated by all stakeholders. This would require the development of a more sensitive and practical detection method.

Without the implementation of long-term management programs, CWD poses a real threat to both wildlife and human health. This can be observed by analyzing both experimental models and real-world data. Gross^16^ noted that mechanistic models of CWD simulations failed to produce stable coexistence of CWD once established in mule deer populations. Similarly, observations of real-world data on the effects of CWD prevalence in the white-tailed deer population showed significantly lower survival rates^15,17^. Deer, specifically white-tailed deer, play important roles culturally and economically and serve as a potential food source^18,19^. A threat to deer populations is not only an ecological threat with unforeseeable effects but also a threat to the human elements that are interconnected with their populations. To compound the issues regarding the possible effects on cervid populations, CWD prions have shown zoonotic potential, which poses a serious human health concern^20,21^. Based on in vitro studies, CWD prions have zoonotic potential. However, human infection by this prion has not been reported. Thus, testing human patients is not necessary. Barria^20^ demonstrated that CWD is susceptible to the human conversion of PrP^C^ to PrP^RES^ using experimental models. Specifically, while barriers exist for prion diseases, such as scrapie in sheep, which prevent the conversion of human prion proteins, no such barrier exists for the CWD protein^17^. These observations highlight the need for more accurate management techniques that require more sensitive detection methods.

Detection methods, whether considering an emergent or an established disease, have been essential to the control of disease^22^. Among the various detection methods available, 4 tests remain the best options. The first is the immunohistochemistry (IHC) assay, which detects PrP^RES^ in neurologic tissue^23,24^. IHC, in combination with an enzyme-linked immunosorbent assay (ELISA), which measures the substance in question via the binding of antibodies and antigens, is considered the gold standard in CWD testing (maintaining an overall agreement of ≥95.7% - ≥97.6%)^25,26^. Another test has been developed and is named protein misfolding cyclic amplification (PMCA). This test stimulates prion replication^27^. Recently, detection methods have evolved to include real-time quaking-induced conversion (RT-QuIC), which provides ample substrate for PrP^SC^ conversion, vigorous shaking and detection of the fluorescent dye intercalated into the newly converted PrPSc^6^. These technologies are still in various stages of development and are not currently used for CWD diagnosis for disease management. Table 1 is a performance table listing the detection of other types of prions or proteins.

**Table 1:** Different testing types for prions or proteins

Sensing Scheme	Sensitivity (nM)	Range	Advantages	Drawbacks	Ref
**Surface-enhanced Raman scattering (SERS)**	~10^2^ protein		Selectively probe for specific regions or domains of a protein	Substrate Variability	^40,41^
**Electrochemical**	10^–9^	10^–9^–10^3^	Wide Detection Range	Complex Sample Preparation	^42^
**Fluorescence**	0.05	0.05–0.30	Improved Photostability, Simultaneous Detection and Imaging	Complex Experimental Setup	^43^
**Colorimetric**	8	8–200	Wide Detection Range	Limited to rPrP Detection, Potential Cytotoxicity.	^44^
**Chemiluminescence**	4.2 pmol/spot		Inexpensive, and Possibility for Simultaneous detection	Requires specialized equipment. More complex assay setup	^45,46^
**EQCM biosensor based on carbon nanotubes**	0.048	0.1–3	Fast Response Time	Manufacturing Complexity, Potential Cytotoxicity	^47–49^
**Resonant mirror biosensor based on plasminogen as a recognition element**	2.4		Real-time Monitoring, High sensitivity	Limited Range of Target Molecules, Potential Cross-reactivity	^50^

There is a clear need for a more advanced and yet practical detection method. PMCA has been noted to be hindered by technical difficulties^28^. IHC is specific but not highly sensitive^29^. In contrast, ELISA is known for its sensitivity. Even RT-QuIC, which is among the most sensitive tests available (solving previous issues regarding limited sensitivity), also has some limitations^30^. Currently, RT-QuIC takes 40–50 hours or more to complete a run^14^.

In the search for other testing techniques to provide a solution to detect pathologic prions, the concept of using electrochemical and optical sensing techniques are possible solutions to the general problem of early detection. The electrochemical sensing technique has been extensively investigated, including many scientific papers, with excellent sensitivity, specificity, and detection in < 1 hour^31^. For example, the addition of nanostructured materials and the use of microfluidic channels have been used to improve the limit of detection (LoD)^32^. Another group demonstrated a new platform where an *E. coli* whole cell based on cell agglutination was utilized for biosensing, with a surface displaying nanobodies to selectively target the analyte^33^. Biosensors based on impedance were used to detect *Salmonella* in water sources with an LoD of 3 CFU/mL^34^. In another study, an immunosensor based on screen-printed IDEs and wheat germ agglutinin was used to detect *E. coli O157:H7* with an LoD of 10^2^ CFU/mL^35^. Optical-based biosensors have been extensively studied^36^. For example, surface-enhanced Raman spectroscopy (SERS)-based sensing was used to distinguish ZIKV biomarkers^37^. This sensor has many advantages, including high sensitivity and selectivity and low cost. However, SERS sensors require the use of an expensive Raman system and laser source, causing difficulty to build a portable system, and its operation would require personnel with some expertise. Paper-based lateral flow assays (LFAs) were used to detect antigens within minutes based on a sandwich immunoassay^38^. Although LFAs have demonstrated good sensitivity, specificity, reproducibility, and assay stability, there is still room for improvement.

Due to the current testing limitations, a more sensitive, real-time, field-deployable detection method with the potential for reliable and practical testing (including and especially antemortem testing) is needed; a newly developed method could have an enormous positive impact on the surveillance and scientific understanding of the disease. Notably, none of the CWD tests described above actually detects the physical shape of the misfolded prion protein. The detection method developed and covered in this paper aims to detect the physical differentiation of misfolded prion proteins.

## Materials And Methods

### Biosensor design and modeling

The biosensor utilizes positive dielectrophoresis (pDEP) to concentrate and trap the CWD prion protein on top of the detection region. DEP refers to the movement of dielectric particles, cells, or proteins in a suspending medium when subjected to a nonuniform AC electrical field (E-field). The direction of movement is determined by the relative permittivity of the particle/protein and the surrounding medium, as well as the frequency of the applied E-field. In our project, we adjusted the AC voltage and frequency to guide the CWD prion proteins toward the centerline region with a high E-field intensity and gradient. The concentrated sample or fluid containing the CWD prion protein was guided to flow toward the detection channel. On the other hand, the bulk fluid that did not contain the CWD prion protein was directed to the outer channels, leading it to the waste outlet. This pDep mechanism ensured the detection of low concentrations of prions.

Similarly, the trapping electrode pairs use positive pDEP to stop and capture the CWD prion protein on the top surface of the detection region. This mechanism efficiently maximizes the captured prion protein or antigen, leading to an increased concentration and ultimately improving the limit of detection (LOD). By utilizing pDEP, the biosensor achieves enhanced sensitivity, enabling reliable detection and analysis of the target protein or antigen at low concentrations. The DEP forces acting on a homogeneous spherical particle can be described by the following equation:

$${F}_{{DEP}}=2\pi {\varepsilon }_{m}{r}^{3}\nabla {E}^{2}{\rm{Re}}[K\left(\omega \right)]$$

where r represents the radius of the sphere, ${{\boldsymbol{\varepsilon }}}_{{\boldsymbol{m}}}$ is the complex permittivity of the medium, E is the magnitude of the applied E-field, and ∇(E^2^) represents the gradient of the squared E-field. The Clausius-Mossotti (C-M) factor, K(ω), determines the direction and strength of the DEP force. By adjusting the frequency of the applied E-field, the value of K(ω) can be modified to generate p-DEP, which attracts the cells toward the center of the focusing and trapping regions. Additionally, due to the ramp-down shape of the channel, the hydrodynamic force also contributes to pushing the cells toward the center of the focusing region and directs the flow toward the detection region.

**Fig. 1:**
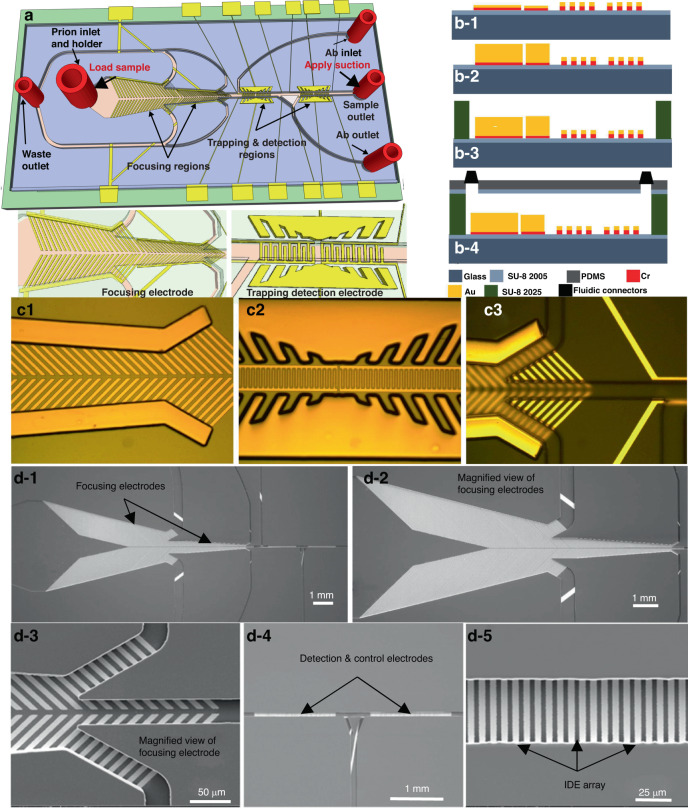
Description of the Biosensor. **a** 3-D view, **b1**–**b4** sideview. **c1**–**c3** Optical images of the biosensor after fabrication of the focusing electrode pair, the sensing and trapping electrodes array, and the SU8 microchannel. **d1**–**d5** Scanning electron microscope (SEMs) micrographs of the fabricated biosensor. **d-1** The two set focusing electrodes, detection electrode, and control electrode embedded in SU-8 microchannel, **d-2** a magnified view of the two-set focusing electrode, **d-3** magnified view of one focusing electrode, **d-4** detection and control electrodes, **d-5** a magnified view of the detection IDE array

**Fig. 2:**
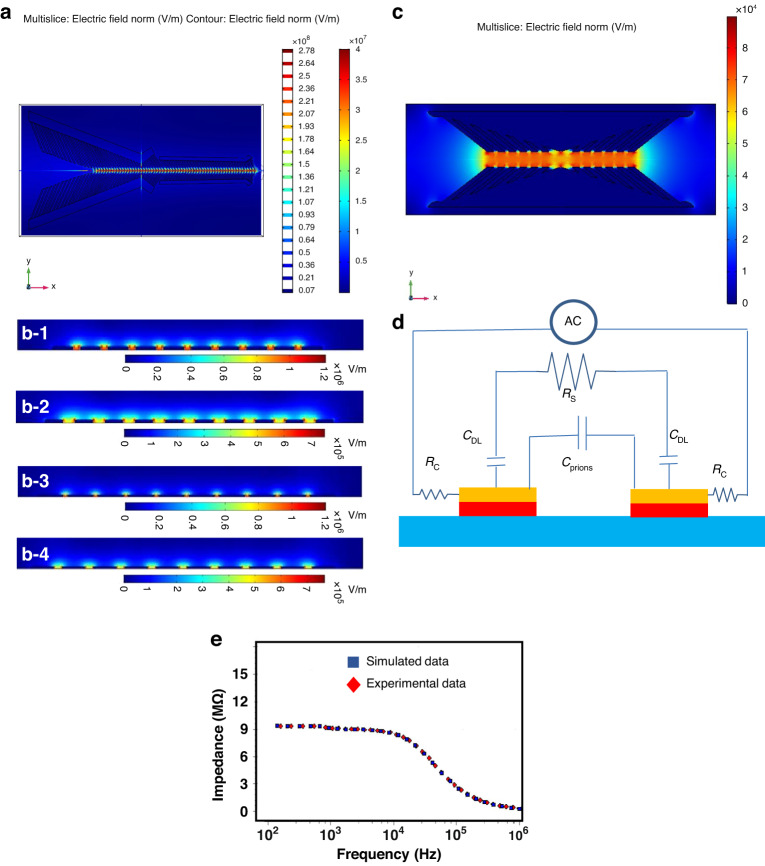
Modeling and Simulation of the Electric field. An electric field (E-Field) modeling and simulation using COMSOL Multiphysics software of the three regions making the biosensor, i.e., **a** focusing. **b1**–**b5** Detection IDE array. The finger width and spacing between fingers are **b-1** 5 µm and 2 µm, **b-2** 4 µm and 4 µm, **b-3** 10 µm and 2 µm, **b-4** 10 µm and 4 µm, respectively. **c** Trapping. **d** An equivalent electrical circuit of the biosensor, **e** experimental and simulation results after the CWD prion antibody-antigen binding in the microchannel

We designed an impedance-based microfluidic microelectromechanical system (MEMS) biosensor with unique features; these features significantly improved its ability to focus and concentrate low quantities of CWD pathologic prions in retropharyngeal lymph nodes to a detectable level and trap and capture and detect CWD prions with high sensitivity and selectivity using arrays of interdigitated electrodes (IDEs) coated with specific antibodies. The biosensor design included three novel regions/functionalities, as shown in Fig. 1a: (1) To concentrate the prion protein effectively, a dedicated region was developed. This region featured two sets of focusing electrodes connected in parallel fashion in a single horizontal microfluidic channel. Each electrode set consisted of a thick ramp-down electrode made of electroplated gold, along with 45° tilted finger pairs made of Cr/Au thin films within a ramp-down channel. This unique combination generated p-DEP forces that efficiently focused and concentrated the prion antigen at the center of the channel and were directed to flow toward the detection microchannel. Moreover, the bulk fluid, which was over 90% of the volume of the original sample media but free of the prion protein, flowed toward the outer channels and was directed to the waste outlets. The biosensor’s distinct design elements, including the ramp-down electroplated vertical sidewall and tilted thin film fingers, created a strong E-field intensity and gradient. This gradient effectively propelled the prion antigen toward the channel’s centerline where the E-field intensity and gradient were highest, regardless of its position across the channel’s width or height. By incorporating this concentration region, the biosensor significantly enhanced the detection sensitivity of CWD prion proteins compared to impedance biosensors lacking a focusing mechanism. The optimal geometry for maximizing the E-field intensity and gradient at the center of the channel was determined through extensive simulations using COMSOL (Fig. 2a). Based on these simulations, the first set of electrodes had a length of 2 mm, while the second set had a length of 4 mm. The spacing between the vertical electrode pair was initially 3.6 mm and gradually decreased to 1.2 mm. Similarly, the spacing between the end points of the electrode pair started at 0.63 mm and decreased to 0.210 mm. The gold (Au) fingers within the electrode pairs had a width of 10 µm, and there was a spacing of 5 µm between the fingers. Additionally, there was a spacing of 10 µm between the finger pairs. The E-field intensity distribution obtained from the simulations revealed a high intensity in the middle of the channel and a significantly lower intensity elsewhere. This optimized design resulted in improved detection sensitivity by effectively concentrating the prion protein in the center of the channel and directing it toward the detection region.

(2) To further enhance the concentration of CWD prion protein/antigen, a dedicated trapping region was incorporated into the biosensor design. This region was composed of vertical electrode pairs, along with vertical fingers made of electroplated gold, which surrounded the detection electrode region. The trapping electrodes leveraged positive p-DEP to generate a high E-field intensity and gradient strong enough to effectively stop and trap the prion antigen on the top surface of the detection electrode. This trapping electrode design ensured that the prion antigen was captured regardless of its position across the width or height of the channel. During the binding process, the trapping electrodes were activated for a 10-minute duration to allow ample time for the interaction between the CWD prion antigen and the immobilized antibodies. By incorporating this dedicated trapping region, the biosensor greatly enhanced its sensitivity compared to impedance-based biosensors lacking this mechanism. This enhancement in sensitivity enabled the reliable detection of CWD prion protein/antigen at low concentrations. COMSOL simulations were employed to optimize the trapping electrode geometry. When a modulated AC voltage was applied across the trapping electrode pairs, a nonuniform E-field gradient is generated. The highest gradient was observed between the trapping electrode pairs across the channel’s width, while the lowest gradient was along the channel’s length, away from the trapping region (as depicted in Fig. 2c). By applying a specific frequency and voltage, the prions or other dielectric particles became polarized, exhibiting p-DEP behavior. Consequently, they were forced to migrate and stop on top of the detection electrode. Through the incorporation of the focusing and trapping regions, the biosensor achieved enhanced sensitivity, thereby facilitating reliable and accurate detection of CWD prion antigen. Based on these simulations, the electrode had a length of 1151 µm, while the gold fingers within the electrode pairs had a width of 30 µm, and there was a spacing of 30 µm between the fingers. Additionally, there was a spacing of 10 µm between the finger pairs. The detection channel height was 55 µm.

(3) To maximize the detection sensitivity, the region designated for prion detection utilized two sets of interdigitated electrode (IDE) arrays. One array was specifically designed for detection purposes, while the other array served as a negative control. This region was situated within a microfluidic channel with a width of 50 μm, which was notably narrower than the focusing region. To achieve optimal sensitivity, the geometry of the detection electrode was thoroughly modeled and simulated using COMSOL (Fig. 2b). The simulations involved systematic variations in finger width and spacing between the fingers, following these configurations: (a) 5 µm width and 2 µm spacing, (b) 4 µm width and 4 µm spacing, (c) 10 µm width and 2 µm spacing, and (d) 10 µm width and 4 µm spacing. Based on these simulations, the electric field (E-field) intensity was notably higher in cases (a) and (c), where the spacing between the fingers was 2 µm. This finding indicated that the spacing between the fingers had a greater influence on the strength of the E-field intensity compared to the width of the fingers. However, fabricating a spacing of 2 µm produced significant challenges. Consequently, the decision was made to fabricate our biosensor with a finger width of 5 µm and a spacing of 3 µm.

In the initial setup, one electrode was coated with a specific antibody-cross linker mixture. This coating process occurred via the antibody inlets while ensuring that neither the control electrode nor the focusing electrodes were contaminated. By avoiding the application of the antibody to the control electrodes or focusing electrode, the biosensor enabled highly sensitive detection of the prion antigen based on impedance change. The second electrode functioned as a negative control and remained uncoated with the antibody. To facilitate antibody coating on the designated electrode without contaminating the control and focusing electrodes, a microfluidic channel with precise fluidic flow control was incorporated into the design. This channel enabled controlled flow of the antibody, ensuring that it only reached the intended electrode. Notably, the IDE array alone, with or without the microfluidic channel, was insufficient to achieve the required sensitivity for prion detection. The inclusion of focusing and trapping regions into the biosensor design was essential to achieve the sensitivity needed to detect CWD prion antigen.

### Equivalent circuit model

We simulated an equivalent electrical circuit to study the impedance response in the detection region (Fig. 2d). The equivalent electrical circuit included the solution resistance (R_S_) and the double-layer capacitance (CDL) between the electrode and the solution, which was a mixture of the engineered prion antigen from the ELISA kit and distilled water. The RS and CDL were connected in series and were proportional to the solution resistivity (ρ_S_). The electrode impedance response was monitored and simulated when the CWD prion antigen bonded with the corresponding CWD antibody on the surface of the detection electrode. The presence of prions in the solution led to the formation of a dielectric capacitance (C_Prions_), which was connected in parallel with both CDL and RS. The value of C_Prions_ was dependent on the dielectric constant of the solution and the geometry of the detection electrode. The impact of the electrical wiring on the circuit was neglected, as it was considered to be negligible compared to other circuit components. As a result, the total impedance consisted of the resistance (R_S_) and the impedance of the two capacitors (CDL). The solution resistance (R_S_) when an AC voltage was applied is given by the following relationship:

$${R}_{S}=\frac{v}{I}{\rho }_{S}\frac{D}{A}$$

where A is the surface area of the detection electrode, D is the spacing between the interdigitated fingers, and ρ is the solution resistivity. The total impedance is given by the following:

$${Z}_{{Dl}}=2{Z}_{{Dl}}+{R}_{s}$$

$${Z}_{{Dl}}=1/i\omega {C}_{{prions}}$$

$${C}_{{Prions}}=\frac{{\varepsilon }_{r}{\epsilon }_{0}A}{D}$$

where ${\epsilon }_{0}$ and ${\varepsilon }_{r}$ are the vacuum permittivity and the solution relative permittivity, respectively, and *ω* is is an angular frequency (in radians per second). An EIS spectrum analyzer was used to analyze the equivalent electrical circuit response. The values of C_Prions_ and R_S_ were obtained to be 20 nF and 9.6 MΩ for the *CWD prion antigen* with a 1:4 dilution sample. The large value of R_S_ was due to the large number of *CWD prion antigens* that were bonded to the CDW prion antibody on the electrodes.

The results indicated that the resistance of the CWD prion antigen had a greater impact on the impedance value in the low-frequency range, while the dielectric capacitance had a smaller impact. Thus, the amount of CWD prion antigen in the solution was the primary factor that caused changes in the impedance response. The impedance values of the CWD prion antigen were high at low frequency and low at high frequencies. From Fig. 2e, the impedance value was only dependent on the dielectric capacitance at higher frequencies, and the CWD prion antigen had no impact on the impedance value. Hence, a frequency range of 100 Hz to 1 MHz was selected to obtain an acceptable Bode plot.

### Biosensor microfabrication

**Fig. 3:**
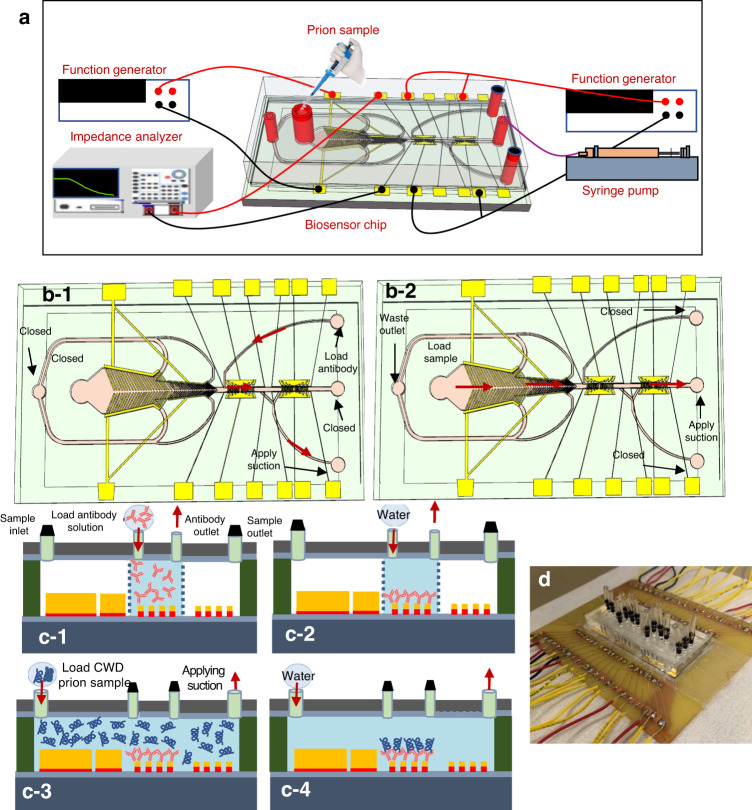
Device Operation. **a** A cartoon showing the experimental setup. Top-view cartoon showing the flow direction during: **b-1** antibody coating where it was first placed at the antibody inlet and suction was applied to the antibody outlet while all other inlets were closed, **b-2** CWD prion antigen loading at the sample inlet while suction was applied to the waste outlet. The flow continued toward the detection region. The process flow for antibody immobilization, and the antibody/ antigen binding on the interdigitated microelectrode: **c-1** the antibody was loaded from the antibody inlet while suction was applied from the antibody outlet, **c-2** the microchannel was washed after adhesion of antibody to the IDE array, **c-3** the CWD prion protein sample was loaded into the sample inlet while suction was applied to the sample outlet, **c-4** the microchannel was washed again after antibody antigen binding was completed, **d** a package biosensor

**Fig. 4:**
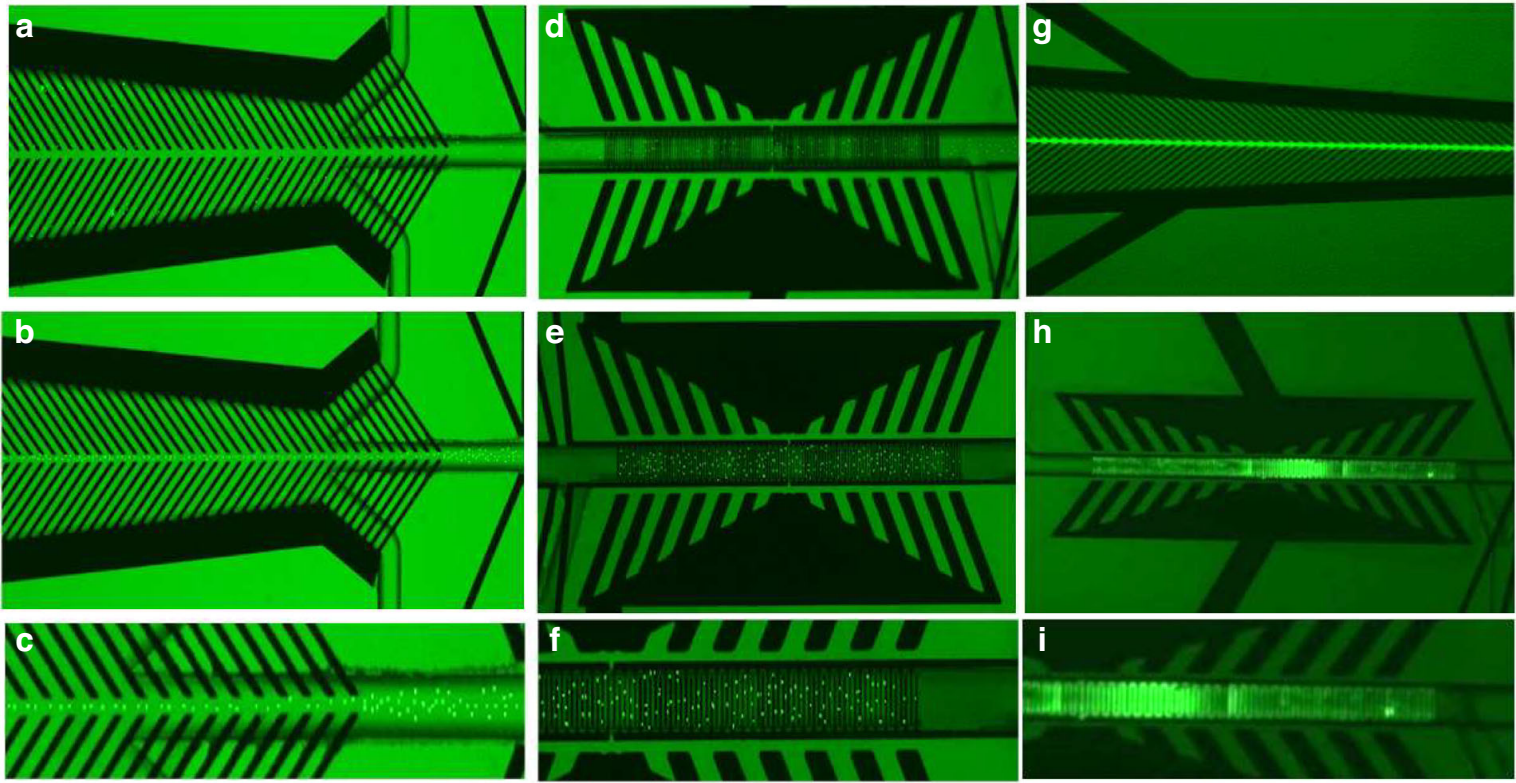
Demonstration of the focusing and trapping capabilities. **a** Fluorescent images before focusing the nanobeads (diameter < 1 μm) into the centerline of the focusing region, **b** fluorescent images after focusing the nanobeads into the centerline of the focusing region. **c** Magnified view of image **b**, **d** fluorescent images after focusing and before trapping the nanobeads onto the surface of the detection electrode array. **e** Fluorescent images after focusing and trapping the nanobeads onto the surface of the detection electrode array. **f** Magnified view of images **e**. **g** Fluorescent images after focusing the nanobeads (diameter < 200 nm) into the centerline of the focusing region, **h** fluorescent images after trapping the nanobeads onto the surface of the detection electrode array, **i** Magnified view of image **h**

The biosensors were micromachined on standard glass substrates as follows (Fig. 1b1–b4). (1) A piranha solution consisting of H_2_O_2_ and H_2_SO_4_ at a 1:3 ratio was used to clean the substrate for 3 minutes to remove organic residues, contaminants, and particles. The substrates were then soaked and flushed with deionized water. (2) To improve the adhesion of the microchannel to the glass substrate, a layer of negative photoresist (SU8-2005) was spin-coated on the substrate, prebaked on hotplates, exposed to ultraviolet light for 12 seconds, and postbaked at the same temperature, i.e., 65 °C and 95 °C for 1 minute and 2 minutes, respectively. This was followed by hard baking of the substrate at 150 °C for 40 minutes to remove the solvent and solidify the photoresist to achieve a thickness of 4 µm. (3) To create the focusing, trapping and detection microelectrodes and the traces and bonding pads, a chromium/gold (Cr/Au) layer was evaporated using an e-beam evaporator. The gold layer was then patterned using Shipley 1805 photoresist and wet etched using a gold etchant (Techni gold 25 ES RTU), which consisted of potassium iodide, iodide, and deionized water. The Cr layer was not etched and was used in the subsequent electroplating step to allow continuous DC current to flow through the substrate and thus enable electroplating gold on all biosensors in the substrate, as shown in Fig. 1b-1. (4) To create the vertical focusing electrode sidewalls as well as the vertical trapping electrode pairs, we patterned a micromold with a thickness of 15 µm using a thick layer of AZ4620 photoresist, which was spin coated, soft baked at 95 °C for 4 minutes, developed using AZ400k, washed with DI water and blown dry with N_2_ (Fig. 1c-1). (5) Gold was then electroplated and filled the micromold using Au electroplating solution (Technic gold 25 ES) (Fig. 1b-2 and c-2). To achieve uniform electroplating, gold electroplating solution was heated at 54 °C and stirred at 75 RPM on a hotplate. To achieve a thickness of 15 µm, 60 nA was applied between the electrode and the platinum mesh counter electrode for 4 hours. Then, after electroplating was completed, the micromold was removed with acetone and cleaned with isopropanol (IPA), and the Cr layer was etched using Cr etchant (Sigma Aldrich). (6) The channel was patterned with a thickness of 55 µm using SU8-2050. After spin coating, the substrate was baked at 65 °C for 2 minutes and 95 °C for 6.5 minutes, UV exposed for 10 seconds, baked again at 65 °C for 1.5 minutes, and 95 °C for 5.5 minutes. It was then developed using PMGA for 1 minute (Fig. 1b-3 and c-3). To harden the SU8, the substrate was baked at 150 °C for 30 minutes. Figure 1 (d) shows scanning electron micrographs (SEMs) of the fabricated sensors. (7) The microchannel/device was covered with a polydimethylsiloxane (PDMS) slab, which was first thoroughly mixed with a curing agent and loaded into a 3-dimensional printed structure specially designed to create a slab with inlets and outlets corresponding to the exact locations of the inlets and outlets on the SU8 layer. The PDMS was cured overnight ( > 24 hours). The PDMS slabs were then exposed to an oxygen plasma, coated with the SU-8 2005 layer, and placed on a hotplate at 100 °C for 20 minutes. It was then bonded to the SU8 microchannel at 50 °C. The bonding was performed on a hotplate and lasted for 10 minutes to improve the strength of the bond between the PDMS and SU8 microchannel. The fluidic connectors were placed at the inlets and outlets and sealed with PDMS and then with glue (Fig. 1b-4 and Fig. 3d. (8) A preprepared printed circuit board PCB with a window and large copper pads was used for wiring with silver paste. A second wire was bonded to the PCB board and connected to an impedance analyzer (Agilent 4294A) for measurements (Fig. 3a).

### Antibody preparation

Anti-prion monoclonal antibody (mAb) (F99/97.6.1, VMRD) and anti-bovine coronavirus (BCV) mAb (5A4, Bio-Rad) were obtained commercially and diluted with PBS according to the manufacturers’ instructions to achieve the desired concentrations for testing. To enhance the adhesion of mAb to electrodes, equal volumes (150 µL) of mAb and sulfosuccinimidyl 6-[3-(2-pyridyldithio) propionamido] hexanoate (sulfo-LC-SPDP) serving as a cross linker were mixed and incubated at room temperature for 1 hour. Subsequently, 100 µL of a reducing buffer containing 200 mM dithiothreitol (DTT), 0.1 M sodium acetate, and 0.1 M NaCl at pH 4.5 was added to the mixture and incubated at room temperature for 30 min^39^. The antibody cross-linker mixture was then loaded and immobilized on the first electrode array without contaminating the control electrode (second set).

### Antigen preparation

The CWD positive control antigen included in the CWD ELISA kit (IDEXX cat# 99-09663) was ready to use. The antigen is normal bovine brain tissue homogenate altered to be suitable for CWD prion ELISA, a test based on antigen and antibody interactions. The technical procedures used to create this antigen and the concentration of the antigen are proprietary information and not available to this study. The antigen was diluted with distilled water to 1:4, 1:8, 1:16, and 1:24. The diluted antigen preparations were subjected to biosensor analysis and ELISA testing. In addition, Bluetongue (BT) virus and Epizootic hemorrhagic disease (EHD) virus (pathogens of cervids) were obtained from the Missouri Veterinary Medical Diagnostic Laboratory (VMDL) and used as negative antigen controls. The concentration of BT and BT viral solutions was 10^4^ TCID_50_/mL. These viruses were selected because they are pathogens of white-tailed deer, and their presence in the samples may cause nonspecific reactions.

### Clinical sample preparation

Retropharyngeal lymph nodes (RLNs) were collected from hunter-harvested, sick, found dead, and roadkill white-tailed deer by the Missouri Department of Conservation. The diagnosis of CWD was performed using the CWD ELISA kit by VMDL. In brief, RLN tissue was cut into 3-dimensional sector-shaped sections from the center of the lymph node comprising the largest surface area. A sample, weighing between 0.25 − 0.35 gr, was minced into 8 − 10 smaller pieces using a scalpel, placed in tubes containing ceramic beads with 0.9 mL distilled water, and then homogenized using MagNA Lyser for 2 run cycles of 50 seconds at a speed of 6.5 m/s. The tissue homogenates were centrifuged at 15,000 RPM for 20 minutes, and the supernatant containing prions was collected and subjected to ELISA testing according to the manufacturer’s instructions. Seven positive and 2 negative RLN samples were made available to validate the biosensor.

To determine the relative limit of detection, an RLN sample was serially diluted from 1:10 to 1:10,000. One hundred microliters of each dilution was subjected to biosensor analysis. To confirm the testing accuracy, the same experiment for each sample at each dilution was repeated three times. To note, the negative samples were not diluted. To verify the detection of CWD pathogenic prion by the biosensor and rule out potential matrix effects in the measured impedance, ELISA-positive RLN samples, including 2 strong positives, 2 medium positives, and 2 weak positives, were subjected to proteinase K treatment. Positivity was determined based on the ELISA OD reading of each sample. For proteinase digestion, 100 µL of tissue homogenate was mixed with 5 µL of proteinase K (Thermo Scientific™ EO0491) and incubated at 56 °C for 1 hour to digest nonprion proteins in the sample. Following digestion, the samples were incubated at 95 °C for 10 minutes to inactivate proteinase K. The treated samples were stored at −20 °C prior to biosensor analysis as described above.

### Experimental testing setup

To test the impedance-based microfluidic biosensor, we initially coated one set of IDE arrays with a mAb (F99/97.6.1)-cross linker mixture. The mixture was placed at the antibody inlet while suction was applied to the antibody outlet, and all other inlets were closed, as shown in Fig. 3b-1, b-2. Antibody suction from the antibody outlet was performed using a Harvard Apparatus syringe pump (PHD 2000) for 2 minutes until the flow started to show at the waste outlet, which indicated that the detection channel was filled with solution. At this point, we stopped the flow for 60 min to enable the antibody to adhere to the Au IDE array. This was followed by washing the microchannel with water to remove contaminants or any unbound antibodies, as shown in Fig. 3b-2. The antibody impedance (baseline impedance) was measured using an impedance analyzer (Keysight E4990A) from 100 Hz to 10 MΩ. The impedance testing setup is shown in Fig. 3a. To confirm the accuracy of the measurements, the same experiment for each tested sample and concentration was repeated 3 times. The biosensors were treated as disposable devices, and each device was used only once.

We then tested the engineered prion antigen and known positive and negative RLN samples. The prion concentration in the control antigen was not provided by the manufacturer for proprietary reasons. Each testing antigen or sample was placed at the sample inlet, and suction was applied at the sample outlet to cause the flow of the sample toward the focusing region (Fig. 3b-2 and c-3). A function generator (Keysight E4990A) that was connected to the electrode pairs was turned on with an optimized AC voltage at a specific frequency to produce p-DEP forces that pushed the prion proteins toward the middle of the focusing channel. The fluid that was free of prion continued to flow closer to the sidewall and exit via the waste microchannels toward the waste outlets. The prion-enriched solution then entered the sensing/trapping region/microchannel. The trapping electrode pairs were already connected to a second function generator, which was turned on with an AC signal of 5 Vp-p at 6 MHz to produce p-DEP forces to maximize the number of trapped prions on the IDE array. At this point (a couple of minutes after filling the channel), the syringe pump was turned off to stop sample flow for 0.5 hours, while the function generator was still on to maximize the number of captured prions and enable them to bind to the anti-prion mAb. After an incubation of 30 minutes, the microchannel was washed with distilled water to remove the unbound prions, as shown in Fig. 3c-4). The impedance was measured again between 100 Hz and 10 MHz. To determine the prion impedance alone, the antibody impedance was subtracted from the overall value of impedance. An inverted microscope equipped with a CCD camera was used to observe the microchannel during testing.

## Results And Discussion

### Testing the focusing electrode

The biosensor’s ability to concentrate the CWD prion protein was tested using fluorescent dielectric latex nanobeads (FluoSpheres Carboxylate-Modified Microspheres, Thermo Fisher Scientific) with two sizes. In the first experiment, nanobeads with a diameter of 200 nm were suspended in water, and in the second experiment, nanobeads with diameters < 1 µm were also suspended in water as well. A fluorescence microscope (Leica TCS SP8) equipped with digital light-sheet and diode lasers was used to observe the fluorescent nanobeads from the backside during the focusing and trapping experiments. The beads were loaded into the sample inlet, while suction was applied to the waste outlet. At the same time, the focusing and trapping electrodes were turned on for 30 seconds. An optimum AC signal of 4 Vp-p at 5 MHz was applied to the focusing electrode to cause the nanobeads to move toward the centerline of the microchannel. The voltage and frequency were experimentally determined. The fluorescent images of the beads before and after focusing are shown in Fig. 4a–f. The concentrated bead solution then entered the sensing/trapping microchannel, where another AC signal of 5 Vp-p at 6 MHz was used to bias the trapping electrode, generating positive dielectrophoresis, which stopped and trapped the nanobeads on the top surface of the detection electrode. The fluorescent images of the nanobeads before and after trapping are shown in Fig. 4g–i. These images demonstrated that the nanobeads were perfectly aligned in the centerline of the microchannel, concentrated, and stopped on top of the detection IDE array. The same optimum voltages and frequencies were used to test the CWD prion protein. We used a similar AC signal with the prion protein. Although this voltage may/may not provide an optimum value for the CWD prions protein, it should be fairly close due to the similarity of the relative permittivity.

### Optimization of antibody concentration

**Fig. 5:**
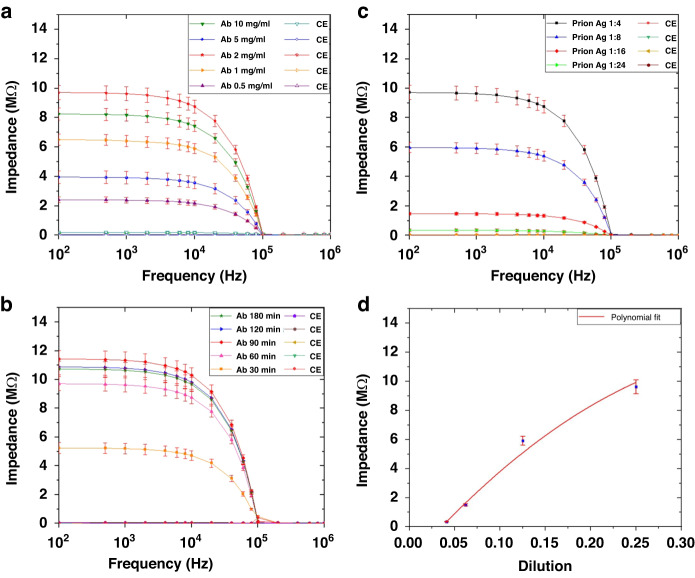
Sensitivity measurement. This includes detection of **a** Different antibody concentrations from 0.25 µg/mL to 10 µg/mL against CWD prion antigen (Ag). **b** Different antibody (Ab) coating time at a fixed Ab concentration of 2 µg/mL (optimum value). **c** Prion antigen from the ELSIA kit at different dilutions. **d** Detection of CWD Prion antigen as a function of concentration (1:4, 1:8, 1:16, 1:24) at 100 KHz. The data was fitted with a polynomial. This shows that the impedance change was not linear

To determine the optimal mAb (F99/97.6.1) concentration, we prepared multiple antibody dilutions from 0.25 µg/mL to 10 µg/mL, and the mAb was then mixed with a cross-linker (Sulfo-LC-SPDP). Each antibody concentration/dilution was tested using a fixed concentration (1:4 dilution) of the control prion antigen sample and a fixed antibody coating time, i.e., 60 minutes. The microchannel was cleaned after antibody-antigen binding using DI water for 15 minutes, and the antibody impedance was tested and recorded. Then, a control prion antigen sample at a dilution of 1:4 was placed at the sample inlet, and suction was applied at 1.5 µL/min from the sample outlet, causing the sample to flow toward the focusing electrode. The prion antigen was concentrated and filled the trapping-detection regions for 30 minutes, as described in the experimental setup section. The microchannel was then cleaned with distilled water for 15 minutes, and the impedance was measured again and subtracted from the antibody impedance; the changes were plotted versus frequency while the concentration of the antibody was varied from 0.25 µg/mL to 10 µg/mL (Fig. 5a). The highest impedance signal was achieved with an optimal antibody concentration of 2 µg/mL, which was not the highest concentration. This result indicated that increasing the antibody concentration to increase the binding between the antibody and antigen for the purpose of increasing the impedance signal was not necessary. The use of this smaller antibody concentration was advantageous because it lowered the overall cost of the sensor. Therefore, all subsequent experiments used an Ab concentration of 2 µg/mL.

### Optimization of antibody coating time

To determine the optimal antibody coating time, we performed antibody immobilization on the detection IDE array using anti-prion mAb (2 µg/mL) mixed with a cross-linker at a constant prion antigen concentration of a 1:4 dilution and varied the antibody coating times. As described in the materials and methods section, the mAb-cross linker mixture was loaded into the device and kept on sensing electrodes for multiple time periods (0.5–3 hours), which allowed the adherence of mAb to the detection electrodes. mAb adherence was followed by a cleaning step (15 minutes), measurement of impedance value, and then loading of the prion antigen (1:4 dilution). The antigen was kept on the electrodes for 30 minutes to enable the binding of antigen to mAb to take place. The microchannel was then cleaned with DI water, and the impedance was recorded again. The impedance change was plotted as a function of frequency for different antibody coating times from 0.5 to 3 hours, as shown in Fig. 5b. The optimum antibody coating was achieved in 1 − 1.5 hours. Incubation times longer than 1.5 hours did not show any considerable increase or decrease in the impedance value. The slight increase in impedance change signal for antibody coating time (time > 1.5 hours) did not justify using longer coating time. In contrast, a short incubation time (0.5 hours) yielded a much weaker impedance signal than that of the 1 −1.5 hour incubation. Therefore, the antibody coating time was set to 1 hour.

### Sensitivity of the Biosensor

To determine the sensitivity, an engineered prion antigen from the ELSIA kit was serially diluted (1:4 to 1:32). Each dilution was flowed into the biosensor with the first IDE array coated with anti-prion mAb (F99/97.6.1) at a concentration of 2 µg/mL. The second set of electrodes (the control electrodes) was not coated or contaminated with mAb. The impedance changes across the detection electrode array and the control electrode array were measured from 100 Hz to 10 MHz using an impedance analyzer. As shown in Fig. 5c, the prion antigen impedance was very high when it interacted with the anti-prion mAb, whereas the control impedance was very low, which confirmed that the biosensor could be used for the detection of CWD prion. In addition, the biosensor generated positive signals when the positive control antigen was diluted as low as 1:24 times. The measured background impedance change was < 0.01 MΩ, while the impedance change at a 1:16 dilution was approximately 1.47 MΩ, and the impedance change at a 1:24 ratio was 0.35 MΩ, which was distinguishable from the background impedance change. In addition, we plotted the CWD prion antigen impedance as a function of dilution (1:4, 1:8, 1:16, 1:24) at 100 kHz (Fig. 5d). The data were fitted with a polynomial. The figure shows that the impedance change was not linear. Notably, the diagnosis of CWD prion is primarily qualitative rather than quantitative, and when animals test positive, they are usually removed from the population. Additionally, quantitative testing requires the utilization of specialized reagents that are not readily accessible in the US. This problem will be addressed when the reagents become available. The IDEXX positive control on ELISA testing is shown in Table 2. The results demonstrated that the sample was positive when the dilution was 1:2, 1:4, and 1:8. For lower dilutions, the sample was not detectable. Table 3 compares the performance of the biosensor against the current gold standard ELISA. These results demonstrated that the impedance sensor was more sensitive than ELISA.

**Table 2:** The IDEXX positive control on ELISA testing

Dilution	O.D.	Status
**1:2**	2.479	Positive
**1:2**	1.850	Positive
**1:4**	0.722	Positive
**1:4**	1.065	Positive
**1:8**	0.441	Positive
**1:8**	0.436	Positive

**Table 3:** Comparison of the performance of the biosensor against the current gold standard ELISA

Sample	ELISA				Sensor			
	Dilution				Dilution			
	10^0^	10^–1^	10^–2^	10^–3^	10^0^	10^–1^	10^–2^	10^–3^
**20162145**	+/+	+/+	-/-	-/-	+/+/+	+/+/+	+/+/+	+/+/+
**20095644**	+/+	+/+	-/-	-/-	+/+/+	+/+/+	+/+/+	+/+/+
**20069577**	+/+	-/-	-/-	-/-	+/+/+	+/+/+	+/+/+	+/+/+
**20133816**	+/+	+/+	-/-	-/-	+/+/+	+/+/+	+/+/+	+/+/+
**20133835**	+/+	+/+	+/+	-/-	+/+/+	+/+/+	+/+/+	+/+/+
**20117247**	+/+	+/+	+/+	-/-	+/+/+	+/+/+	+/+/+	+/+/+
**20109952**	+/+	+/+	+/-	-/-	+/+/+	+/+/+	+/+/+	+/+/+

**Fig. 6:**
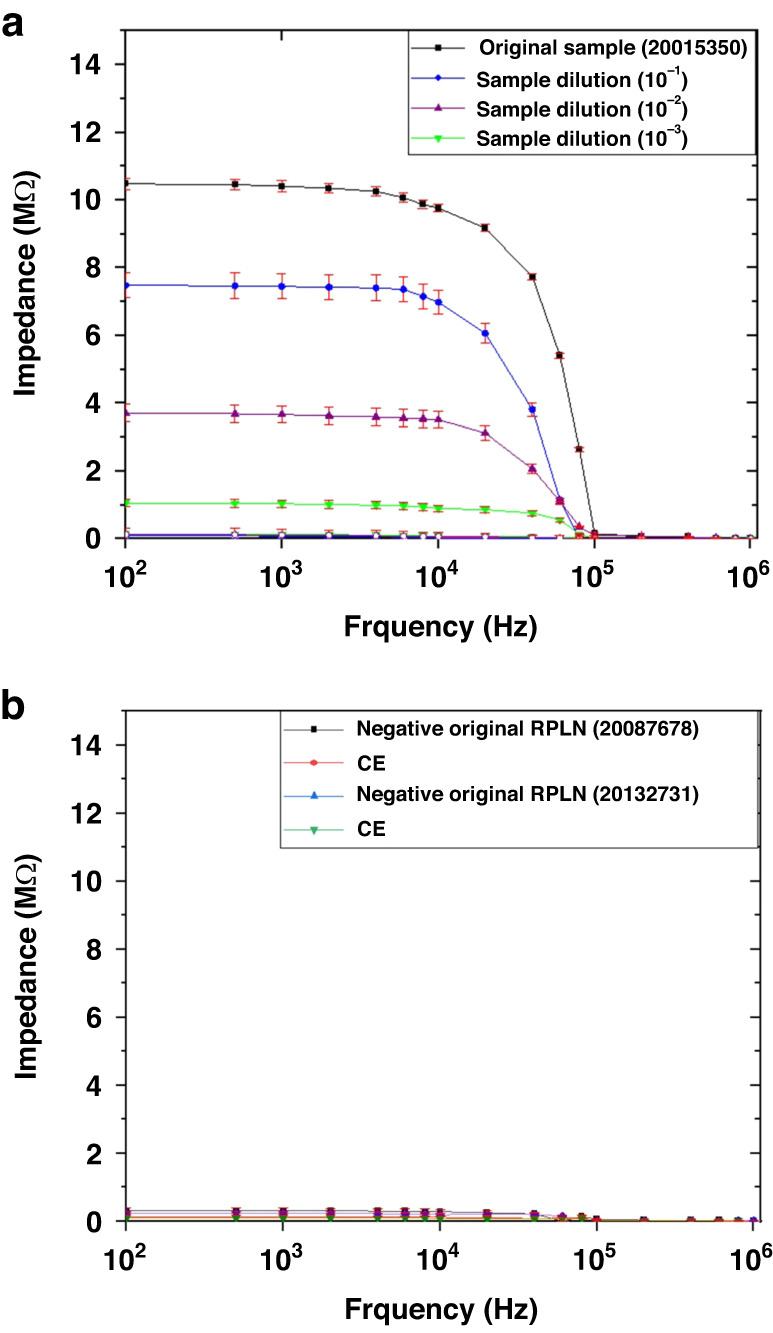
Study of a known CWD positive retropharyngeal lymph nodes (RLN) sample. **a** Relative limit of detection (rLOD) based on a known CWD positive retropharyngeal lymph nodes (RLN) sample (20015350). **b** Known CWD-negative RLN samples

The relative limit of detection (rLOD) was determined by analyzing a known positive RLN sample (20015350). Serial dilutions (1:10 to 1:10,000) of the sample were tested by ELISA and the biosensor. The results (Table 4) showed that ELISA was able to detect the prions at a dilution of 1:100, while the biosensor detected the prion at a dilution of 1:1000 (Fig. 6a). Thus, the rLOD of the biosensor was 1:1000. This result demonstrated that the impedance biosensor was 10 times more sensitive than ELISA. The CWD diagnosis is based on qualitative, not quantitative, testing of samples because all positive animals would be removed. The absolute LOD was not determined because quantitative testing involves special reagents to quantify the CWD prions, and these reagents have not been available for over two years. Two negative RLN samples were also tested in the same manner, but the measured impedance values were very low, comparable to that of antibody alone (Fig. 6b). Notably, the current gold standard for CWD diagnosis is ELISA screening followed by IHC confirmation of ELISA-positive results. Therefore, similar to ELISA, the relative LoD of our impedance-based biosensor is adequate and relevant to the field of CWD diagnostics. The increase by 10-fold in sensitivity (rLoD) is important as the causative agent of the disease, while in the early stage, it is at a lower level in the tissue. This is an important improvement from a biologic and diagnostic standpoint and may help to detect the disease at an earlier stage. Other testing techniques/assays, including PMCA and RT-QuIC, are still in various stages of development, and they are not currently used in CWD diagnosis for disease management. They are based on prion amplification, not antigen-antibody interactions. The current gold standard for CWD diagnosis is ELISA screening of retropharyngeal lymph nodes (RLNs) or Obex samples followed by immunohistochemistry (IHC) confirmation of ELISA-positive results, which are qualitative tests without a published absolute limit of detection. Specifically, a set concentration for CWD detection in clinical testing has not been established due to the complex nature of this prion disease and the need for management programs.

**Table 4:** Serial dilutions (10^–1^ to 10^–3^) of a known positive RLN sample (20015350) were tested by ELISA

Dilution	O.D.	Status
**1:1**	4.256	Positive
**1:1**	4.204	Positive
**1:10**	1.548	Positive
**1:10**	1.361	Positive
**1:100**	0.296	Positive
**1:100**	0.320	Positive
**1:1000**	0.177	Negative
**1:1000**	0.210	Negative

Because determination of prion concentration involves special reagents or mouse bioassays that were not available in this study, we used a relative limit of detection (rLOD) and compared it with the sensitivity of ELISA (Table 2), the most widely used CWD test. Our results indicated that the biosensor is 6 to 10 times more sensitive than ELISA. We did not pursue the comparison of our biosensor with other new technologies, such as PMCA or RT-QuIC, for the following reasons: 1) these assays are still in various stages of development, 2) they are not currently used in CWD diagnosis for disease management, and 3) these technologies are based on prion amplification, not antigen-antibody interactions. Clinically, CWD diagnosis is based on qualitative, not quantitative, analysis of samples because all positive animals would be removed in CWD management programs, and the meat from CWD-positive animals is not able to be consumed by human, as advised by the CDC. The current gold standard for CWD diagnosis is ELISA screening of RLN or Obex samples followed by IHC confirmation of positive ELISA results. Since our goal is to develop a biosensor that is more sensitive, specific, and portable, the rLOD used in this study is adequate and relevant to the field of CWD diagnostics. Further study is underway to determine the biosensor’s diagnostic sensitivity and specificity on RLNs and other types of clinical samples.

We verified that there was no crosstalk in the device by adding the control electrode, where the impedance change was very small (close to zero). Similarly, there was no cross-talk between the focusing electrode and the detection electrode. The antibody did not reach the focusing electrode. Therefore, this eliminated the possibility of cross-talk.

### Specificity and selectivity of the biosensor

**Fig. 7:**
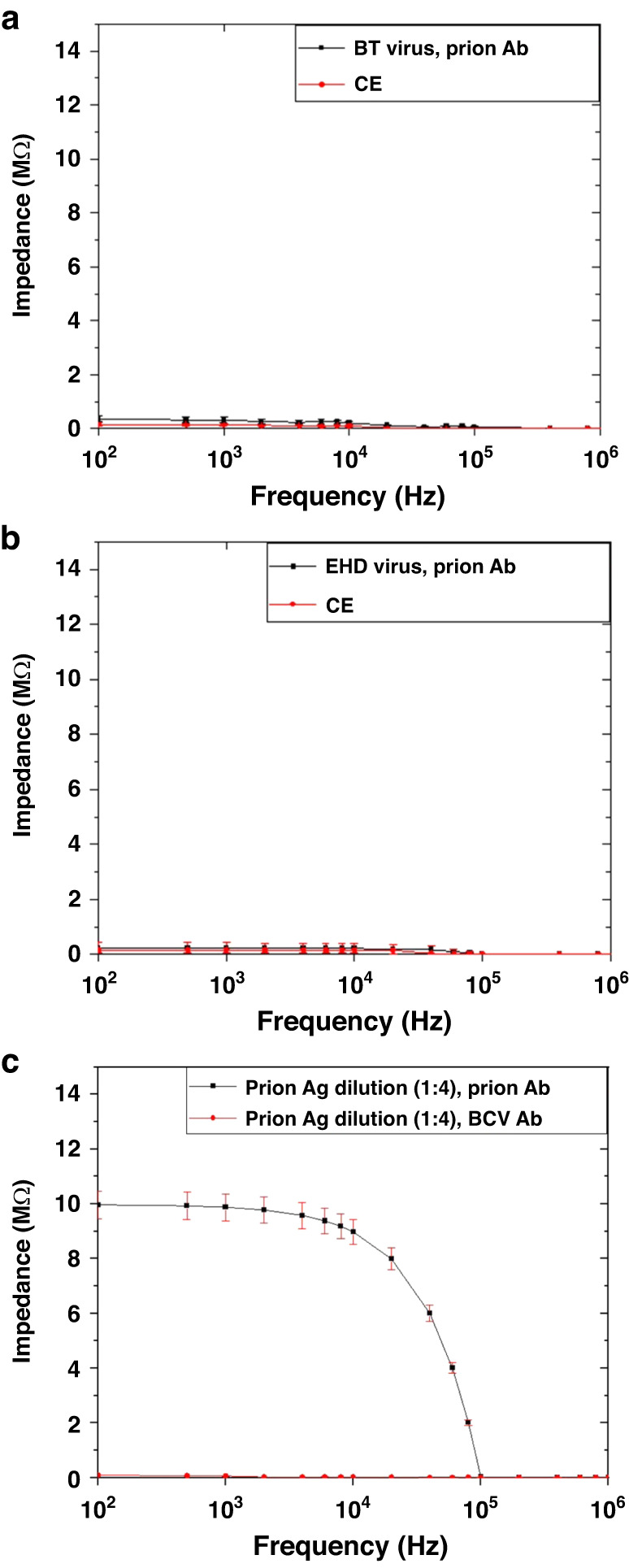
Study of specificity, selectivity, and negative control of the biosensor. **a** Specificity testing using anti-prion antibody and Bluetongue (BT) virus. The negative control electrode was not coated with antibody and exposed to BT virus. **b** Specificity testing using anti-prion antibody and Epizootic hemorrhagic disease virus (EHDV). The negative control electrode was not coated with antibody and exposed to EHDV, **c** Selectivity testing of a known positive RLN sample against anti-prion mAb (high impedance) and anti-BCV antibody (baseline impedance)

To determine the specificity of the biosensor, we tested Bluetongue (BT) virus and Epizootic hemorrhagic disease (EHD) virus as negative control antigens. These viruses were chosen because they are pathogens of white-tailed deer, and their presence in the samples may cause nonspecific reactions. A total of 100 μL of the virus containing 10^3^ TCID_50_ was injected into the biosensor through the sample inlet. Anti-prion mAb (F99/97.6.1) and the cross-linker mixture were loaded into the biosensor. As shown in Fig. 7a and b, loading of these control viruses did not change the impedance measurement values compared to the baseline value for antibody alone. These results demonstrated that the impedance-based biosensor is specific. To evaluate the selectivity of the biosensor, we tested a known positive RLN sample against both anti-prion mAb (2 µg/mL) and anti-BCV mAb (5 µg/mL) antibodies. As shown in Fig. 7c, the measured impedance response for prion antigen/anti-prion mAb was very high, whereas impedance signals resulting from antigen/anti-BCV antibody were near baseline. These results demonstrated that the biosensor was highly selective. The impedance of the control electrodes without antigen was near zero.

### Confirmation of the Detection of Pathogenic Prions

**Fig. 8:**
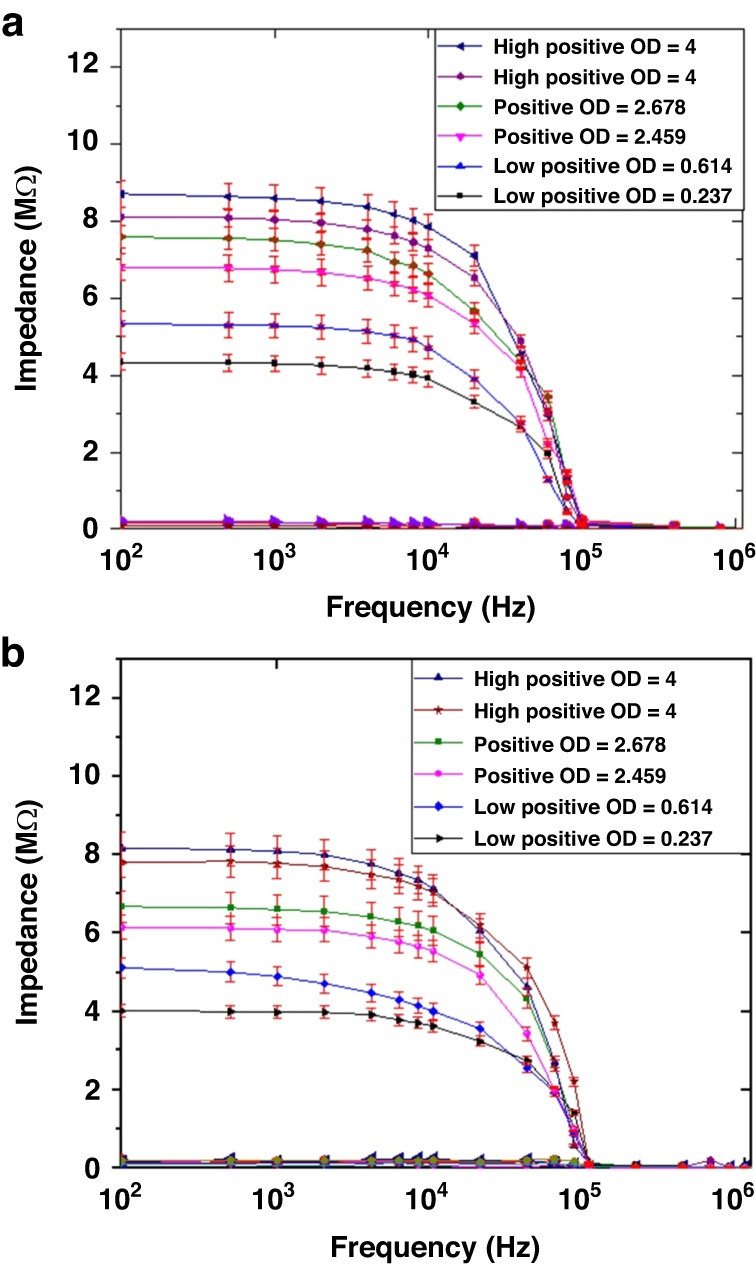
Confirmation of the Detection of Pathogenic Prions. Detection of CWD pathogenic prion in RLNs: includes 2 strong positives (OD = 4), 2 moderate strong positive (OD = 2.678, and 2.459), and 2 weak strong positives (OD = 0.614 and 0.237). **a** Untreated RLN samples and **b** RLN samples treated with proteinase K to remove non-prion proteins in the samples

Pathogenic prions are misfolded proteins that are resistant to proteases. To confirm that the impedance changes or positive signals were indeed generated by the specific binding of pathogenic prions to anti-prion mAb, we treated RLN samples with proteinase K to remove nonpathologic prion proteins in the samples. We selected 2 ELISA strongly positive samples, 2 moderately positive samples and 2 weakly positive samples. The ELISA optical density (OD) of these samples in the order of strong to weak were 4 and 4, 2.678 and 2.459, 0.614 and 0.237. The untreated RLN samples were also tested by the biosensor. As shown in Fig. 7, the impedance values for the untreated RLN samples (Fig. 8a) were similar to the values for proteinase K-treated RLN samples (Fig. 8b), indicating that the impedance changes were caused by the detection of pathogenic prion and that there was no detectable matrix effect.

When prion antigens/proteins bind to anti-prion antibodies, they form large Ag-Ab complexes. The impedance values of the CWD prion antibody (background) were subtracted from the overall impedance to determine the impedance of the prion protein alone in all data/figures presented. The impedance measurement of prion samples from the control antigen from the CWD ELISA kit showed higher impedance values than the real samples. Thus, the concentration of prion was higher in the control samples. When the control samples were diluted more than 1:4, the impedance value reduced, as expected. A proteinase K experiment was conducted to demonstrate that the impedance signal came from pathogenic prions. Specifically, the signal did not include the involvement of other matrix effects, such as nonprion protein. All measurements were repeated at least 3 times to confirm the measurement accuracy. Six tissue homogenates from CWD-positive deer were subjected to proteinase K treatment (details in the manuscript). After proteinase K treatment to remove nonprion proteins, the samples were tested by the biosensor. The impedance values remained comparable to the values for untreated samples, indicating that the impedance was caused by the detection of CWD prion. In all measurements, we used the second electrode as a negative control, which confirmed the accuracy of the measurements. This can be clarified further.

To translate the device into practice in the form of a commercial device, a small portable biosensing system will be built, which can be used to read the signal. The system will include the electrical circuits (e.g., impedance circuit to replace the impedance analyzer), the data analysis software, the hardware, pump, sample and waste holders, and professional-grade data display where the user can enter sample information using a local keypad or keyboard to achieve the full feature (e.g., integration of the biosensor system to smartphone), commercially viable prototype. The biosensor chip is disposable, i.e., one-time use, and can be easily placed and removed from the biosensing system. The user can easily place and remove the biosensor chip into the biosensing system/instrument, load the sample and push the bottom to enable the system to diagnose and indicate the status of the sample, i.e., positive or negative. The disposable device is expected to be manufactured with an approximate cost range from $2.22 to $5.11 depending on the manufacturing volume. More information is provided on this near the end of the discussion.

## Conclusion

MEMS biosensors were developed based on fluidic microchannels and impedance measurements for the rapid detection of pathologic prion protein. Tissues used for the development were lymph node samples from free-ranging deer that had been previously tested using standard laboratory techniques and positive antigen samples from the existing test kit environment. A combination of focusing electrode pairs and trapping electrode pairs was used to maximize the capture of prions to increase sensitivity. One set of IDE arrays was used for the detection of prions, and another set of IDE arrays was used as a control. The biosensor was applied to detect an engineered prion antigen and known positive clinical samples (RLN) at various dilutions and known negative RLN samples from 100 Hz to 10 MHz. To evaluate the specificity and selectivity of the biosensor, nonprion antigens (BTV and EHDV) were tested against anti-prion mAb, and anti-BCV antibody was tested against the prion antigen. Known negative RLN samples and no antigen controls were also included. In addition, the proteinase K-digested RLN samples were tested to confirm the detection of pathogenic prions. The device detected the engineered prion antigen at a dilution of 1:24, while ELISA was able to detect the same antigen at a dilution of 1:8. The biosensor demonstrated a relative limit of detection (rLOD) of 1:1000 dilution of a strong positive RLN sample, while the ELISA rLOD was 1:100. The biosensor also showed high specificity and selectivity as well as the ability to detect pathogenic prions in the RLN samples.
